# Adverse Events of PD-1 or PD-L1 Inhibitors in Triple-Negative Breast Cancer: A Systematic Review and Meta-Analysis

**DOI:** 10.3390/life12121990

**Published:** 2022-11-28

**Authors:** Yixi Zhang, Jingyuan Wang, Taobo Hu, Huina Wang, Mengping Long, Baosheng Liang

**Affiliations:** 1 Department of Biostatistics, School of Public Health, Peking University, Beijing 100191, China; 2 Department of Breast Surgery, Peking University People’s Hospital, Beijing 100044, China; 3 Department of Pathology, Peking University Cancer Hospital, Beijing 100083, China

**Keywords:** triple-negative breast cancer, PD-1 inhibitors, PD-L1 inhibitors, adverse events, safety

## Abstract

(1) Background: This study aimed to develop a comprehensive understanding of the treatment-related adverse events when using PD-1 or PD-L1 inhibitors in triple-negative breast cancer (TNBC). (2) Methods: We conducted a meta-analysis of Phase II/III randomized clinical trials. Studies were searched for using PubMed, Embase, and Cochrane Library from 1 March 1980 till 30 June 2022. Data on adverse events were mainly extracted from ClinicalTrials.gov and published articles. A generalized linear mixed model with the logit transformation was employed to obtain the overall incidence of adverse events across all studies. For serious adverse events with low incidences, the Peto method was used to calculate the odds ratio (OR) and 95% confidence interval (95%CI) in the PD-1 or PD-L1 inhibitors groups compared to the control groups. (3) Results: Nine studies were included in the meta-analysis, including a total of 2941 TNBC patients treated with PD-1 or PD-L1 inhibitors (including atezolizumab, pembrolizumab and durvalumab) and 2339 patients in the control groups. Chemotherapy alone was the control group in all studies. The average incidences of all serious immune-related adverse events of interest (hypothyroidism, hyperthyroidism, pneumonitis, pruritus, rash) were less than 1%, except for adrenal insufficiency (1.70%, 95%CI: 0.50–5.61%) in the PD-1 or PD-L1 groups. PD-1 or PD-L1 inhibitors significantly increased the risk of serious pneumonitis (OR = 2.52, 95%CI: 1.02–6.26), hypothyroidism (OR = 5.92, 95%CI: 1.22–28.86), alanine aminotransferase (ALT) elevation (OR = 1.66, 95%CI: 1.12–2.45), and adrenal insufficiency (OR = 18.81, 95%CI: 3.42–103.40). For non-serious adverse events, the patients treated with PD-1 or PD-L1 inhibitors had higher risk of aspartate aminotransferase (AST) elevation (OR =1.26, 95%CI: 1.02–1.57), hypothyroidism (OR = 3.63, 95%CI: 2.92–4.51), pruritus (OR = 1.84, 95%CI: 1.30–2.59), rash (OR = 1.29, 95%CI: 1.08–1.55), and fever (OR = 1.77, 95%CI: 1.13–2.77), compared with chemotherapy alone. (4) Conclusions: The incidence of serious immune-related adverse events in PD-1 or PD-L1 inhibitors groups is low but significantly higher than in chemotherapy groups. When using PD-1 or PD-L1 inhibitors for the treatment of TNBC, serious pneumonitis, hypothyroidism, ALT elevation, and adrenal insufficiency should be considered. Non-serious adverse events, such as AST elevation, rash, and fever, should also be taken into consideration.

## 1. Introduction

Breast cancer is a noteworthy public health problem, with a rising global burden in many countries [1,2]. Breast cancer is the most common cancer in women, and it ranks first as cause of death [2]. Triple-negative breast cancer (TNBC) is a special type of breast cancer, which refers to the immunohistochemical examination of breast cancer cells showing that estrogen receptor (ER), progesterone receptor (PR), and human epidermal growth factor receptor 2 (HER2) all lack expression [3]. TNBC accounts for 10–20% of all breast cancer patients [4]. However, the prognosis of TNBC is worse than other types of breast cancer. The mortality of TNBC is over 40% within the first five years and most patients develop distant metastasis [5].

Neoadjuvant chemotherapy is a primary pharmacotherapy for TNBC. Because of its negative expression of ER, PR, and HER2, TNBC is not sensitive to endocrine therapy or targeted therapy [6]. The national comprehensive cancer network guidelines recommend using combination regimens based on taxane, anthracycline, cyclophosphamide, cisplatin, and fluorouracil [7]. However, various combinations of chemotherapy drugs may lead to different outcomes and prognoses for TNBC patients. According to available clinical trial results, basal-like 1 subtype TNBC has more sensitivity to chemotherapy than other subtypes, with the highest pathologic complete response (pCR) rate of 52% [8,9]. TNBC has high heterogeneity and lacks useful targets, making it difficult to discover new targets. Despite massive chemotherapy combinations being optional, drug resistance happens inevitably in some patients [5]. Exploring and developing an effective and safe treatment for TNBC is vital. Programmed cell death protein 1 (PD-1) or programmed cell death ligand 1(PD-L1) inhibitors can block the binding of PD-1 receptor protein on the surface of tumor cells with PD-1 receptor in T cells, thereby causing T cells to kill tumor cells [10,11]. The analysis of immunohistochemistry in TNBC patients has discovered that half of the TNBC patients have high expression of PD-1 or PD-L1, which implies that PD-1 or PD-L1 could be a potential target [10,12], while other studies have considered de-glycosylated PD-L1 in TNBC cells as a biomarker [13].

In March 2019, the U.S. Food and Drug Administration (FDA) approved anti-PD-L1 therapy atezolizumab combined with chemotherapy for first-line treatment of patients with PD-L1 positive advanced or metastatic TNBC patients based on the result of IMpassion 130 [14]. This approval made the atezolizumab and abraxane combination the first cancer immunotherapy scheme for the treatment of PD-L1-positive metastatic TNBC. However, in July 2021, Roche withdrew its application to extend the use of atezolizumab to the treatment of TNBC patients in Europe because of the post-market study Impassion 131 [15]. KEYNOTE-012 was the first published result investigating the safety and efficiency of PD-1 inhibitor pembrolizumab in TNBC patients. Pembrolizumab given every 2 weeks to TNBC patients achieved an overall response rate of 18.5% and had an acceptable safety profile [16]. Further studies discovered that platinum-based chemotherapy could make the tumor cell more sensitive to PD-1 or PD-L1 inhibitors by exerting immunomodulation properties [17].

There are several meta-analyses that have evaluated the efficacy and safety of neoadjuvant immune checkpoint inhibitors [18,19,20]. Previously, studies have shown that PD-1 or PD-L1 inhibitors are related to high incidences of various treatment-related adverse events, such as fatigue, pruritus and hypothyroidism [21,22]. In a recent meta-analysis that evaluated the effectiveness of PD-1 and PD-L1 inhibitors combined with chemotherapy for TNBC, researchers found that the combination strategy improved the pCR rate and progression-free survival (PFS) [20], but the combination treatment increased the risk of several adverse events. There are limitations in the previous meta-analysis. Their safety analysis was only confined to three clinical trials (Impassion 130 [23], Impassion 131 [24], and Keynote-355 [25]) and failed to stratify immune checkpoint inhibitor regimens. Thus, it is essential to have comprehensive understanding of treatment-related adverse events using PD-1 or PD-L1 inhibitors in TNBC patients.

In this study, we conducted a systematic review and meta-analysis to thoroughly evaluate the adverse events and the safety of PD-1 or PD-L1 inhibitors in TNBC patients based on extensive randomized clinical trials.

## 2. Materials and Methods

### 2.1. Search Strategy

We systematically searched three databases (PubMed, Embase and Cochrane Library) regarding PD-1 or PD-L1 inhibitors in triple-negative breast cancer from 1 March 1980 to 30 June 2022, independently by two authors (Y.Z. and J.W.). The keywords used in the search strategy were “PD-1”, “PD-L1”, “nivolumab”, “pembrolizumab”, “durvalumab”, “atezolizumab”, “avelumab”, “triple-negative breast cancer” and “TNBC”. We reviewed all the abstracts of the resulting studies and full texts were retrieved.

### 2.2. Study Selection

Three authors (Y.Z., J.W., and H.W.) independently conducted the literature selection. Inconsistencies were resolved by consensus. We used the following criteria for study selection: (1) Patients: triple-negative breast cancer patients, (2) Intervention: using PD-1 or PD-L1 as treatment, including but not limited to monotherapy, (3) Control: we did not make any limitations to the control group, (4) Outcome: the data on adverse events should be reported in the article or website (ClinicalTrials.gov), and (5) published in English. We had the following exclusion criteria: (1) other study designs such as case reports, case series, case–control studies, cohort studies, and so on, (2) protocols and secondary research, such as systematic reviews, pooled analysis, and study protocols, (3) studies that did not focus on PD-1 or PD-L1 inhibitors, (4) animal studies, and (5) duplicates.

### 2.3. Outcome and Data Extraction

We paid close attention to different reported adverse events among TNBC patients treated with PD-1 or PD-L1 inhibitors. The adverse events included anemia, neutropenia, arthralgia, back pain, alanine aminotransferase (ALT) elevation, aspartate aminotransferase (AST) elevation, hypothyroid, pneumonitis, colitis, fever, headache, pruritus, rash, and so on. We first searched ClinicalTrials.gov for the submitted results. For those not available on the website, we extracted data from the published articles. From the data from ClinicalTrials.gov, we extracted both serious and non-serious adverse events. For data from the article, we classified Grade 3 or higher as serious adverse events and Grade 1–2 as non-serious adverse events. Besides the adverse events, we also extracted the author, published year, drug information, and trial name.

### 2.4. Quality Assessment and Data Analysis

We used Cochrane Bias Risk Evaluation Tool to assess six dimensions of bias: randomization process, deviations from intended interventions, missing outcome data, measurement of the outcome, and selection of the reported result. The bias was determined as high risk, low risk and uncertain. After bias assessment, we first conducted a proportion meta-analysis to calculate the overall incidence of serious and non-serious adverse events using the generalized linear mixed model with the logit transformation [26]. For serious adverse events with low incidence, we employed the Peto method to calculate the overall odds ratio (OR) and 95% confidence interval (95%CI) between the treatment group and the control group. We examined the heterogeneity between studies through the Q test and I^2^ statistics. The random effect model was used when there was high heterogeneity (I^2^ > 50%). All data analysis was conducted by R (version 4.1.3).

## 3. Results

### 3.1. Features of Studies

**Figure 1:**
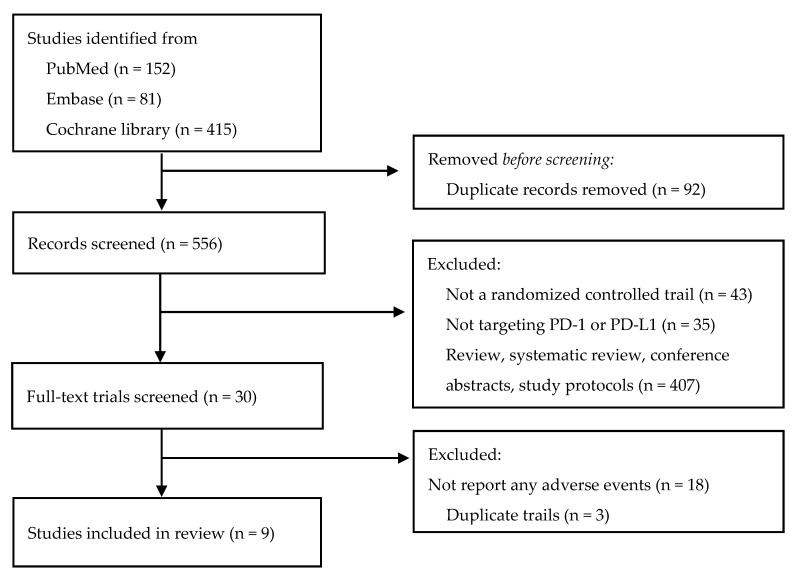
Flow diagram for study selection in the meta-analysis.

We searched 648 studies in total, and 9 studies meeting the selection criteria were incorporated into the research. Figure 1 shows the study selection diagram. Among all the included studies, only one used PD-1 inhibitors [27], three studies used PD-1 inhibitors combined with chemotherapy [25,28,29], and five studies used PD-L1 inhibitors combined with chemotherapy [23,24,30,31,32]. Although we did not limit the control group in the literature search, we found that all the studies used chemotherapy as their control group. As a result, a total of 2941 patients were included in the treatment group, consisting of 1055 patients in the PD-L1 inhibitor atezolizumab group, 1721 in the PD-1 inhibitor pembrolizumab group, and 165 in the PD-1 inhibitor durvalumab group; 2339 patients were in the control group for this meta-analysis. Eight studies were registered on ClinicalTrials.gov and had published their data on adverse events. The data of the remaining study were retrieved from the literature [31]. Table 1 presents information on the included studies.

### 3.2. Risk of Bias Assessment

The risk of bias assessment is summarized in Table 2. We found there was a low bias of selection and outcome. For randomization and deviations, two studies, Pusztai 2021 and Nanda 2020, were considered as high risk. Because the primary aims of the included studies were not related to adverse events, collection of information on adverse events was mainly from online. Thus, we deemed Pusztai et al.’s study [31] at high risk of bias with regard to measurement of the outcome, since its data were from the article.

### 3.3. Meta-Analysis Results

#### 3.3.1. Meta-Analysis Results with Serious Adverse Events

**Figure 2:**
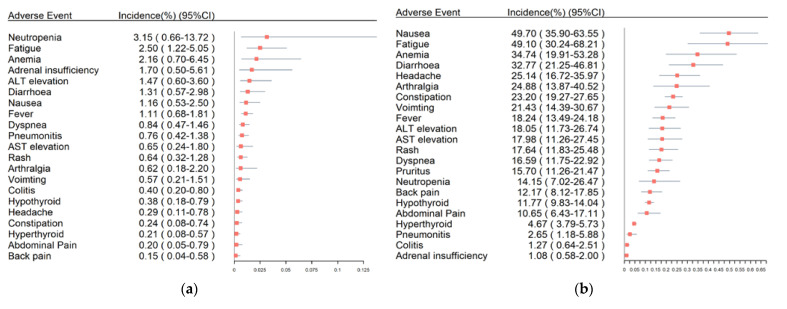
Overall incidences of adverse events in TNBC patients using PD-1/PD-L1 inhibitors: (**a**) incidences of serious adverse events; (**b**) incidences of non-serious adverse events.

The overall incidences of serious adverse events are shown in Figure 2a. In particular, the most common serious adverse events were neutropenia (3.15%, 95% CI: 0.66–13.72%), fatigue (2.50%, 95% CI: 1.22–5.05%), and anemia (2.16%, 95% CI: 0.70–6.45%), followed by adrenal insufficiency (1.70%, 95% CI: 0.50–5.61%) and alanine aminotransferase (ALT) elevation (1.47%, 95% CI: 0.60–3.60%). The incidences of serious immune-related adverse events were lower than 1%, including pneumonitis (0.76%, 95% CI: 0.42–1.38%), hypothyroidism (0.38%, 95% CI: 0.18–0.79%), and hyperthyroidism (0.21%, 95% CI: 0.08–0.57%).

**Figure 3:**
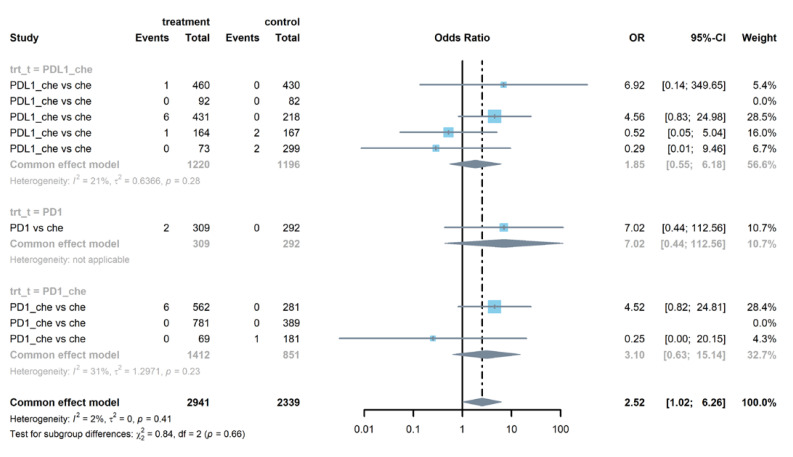
Forest plot of serious pneumonitis in patients treated with PD-1 or PD-L1 inhibitors versus chemotherapy (NOTE: ‘PDL1_che’ means patients treated with PD_L1 and chemotherapy combined therapy, ‘PD1_che’ means patients treated with PD1 and chemotherapy).

**Figure 4:**
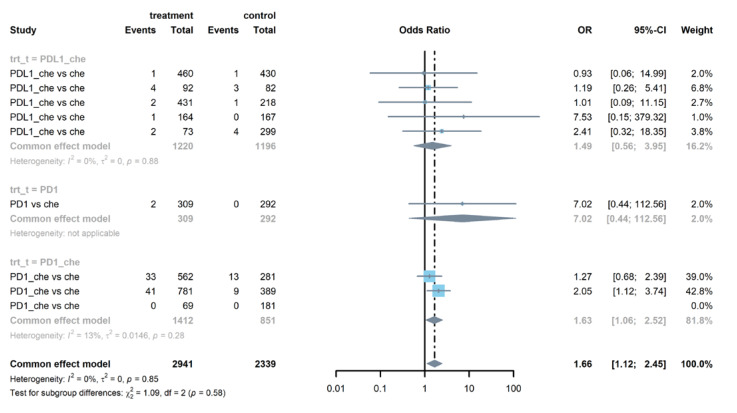
Forest plot of serious ALT elevation in patients treated with PD-1 or PD-L1 inhibitors versus chemotherapy.

Seven studies reported serious pneumonitis and integrated data showed that PD-1 or PD-L1 inhibitors combined with chemotherapy increased the risk of pneumonitis (OR = 2.52, 95% CI: 1.02–6.26), as is shown in Figure 3. There was no heterogeneity (I^2^ = 2%) in the overall meta-analysis. When we separated the PD-1 inhibitors and PD-L1 inhibitors, the results in subgroups were not significant. Four studies reported serious hypothyroidism, from which we found that the PD-1 or PD-L1 inhibitors had higher risk than chemotherapy (OR = 5.92, 95% CI: 1.22–28.86), especially in the subgroup with PD-1 combined with chemotherapy (see Figure S1). Figure 4 illustrates that the ALT elevation had a higher incidence rate in the group using PD-1 or PD-L1 inhibitors than in the chemotherapy group (OR = 1.66, 95% CI: 1.12–2.45). Subgroup analysis indicated that the PD-1 inhibitors group had a higher risk of ALT elevation (OR = 1.63, 95% CI: 1.06–2.52). Figure S2 shows that adrenal insufficiency also showed a significantly higher risk in PD-1 or PD-L1 inhibitors group compared to the chemotherapy group (OR = 18.81, 95% CI: 3.42–103.40).

#### 3.3.2. Meta-Analysis Results with Non-Serious Adverse Events

Figure 2b summarizes the results of non-serious adverse events. The most frequent general adverse events were nausea (49.70%, 95% CI: 35.90–63.55%), fatigue (49.10%, 95% CI: 30.24–68.21%), anemia (34.74%, 95% CI: 19.91–53.28%), diarrhea (32.77%, 95% CI: 21.25–46.81%), headache (25.14%, 95% CI: 16.72–35.97%), and arthralgia (24.88%, 95% CI: 13.87–40.52%).

**Figure 5:**
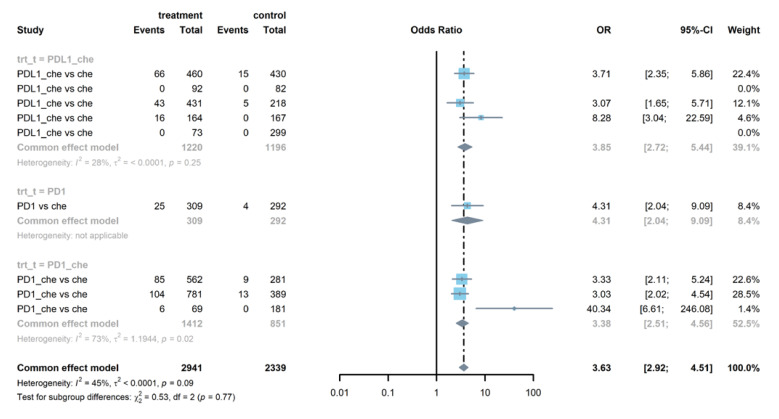
Forest plot of non-serious hypothyroidism in patients treated with PD-1 or PD-L1 inhibitors versus chemotherapy.

**Figure 6:**
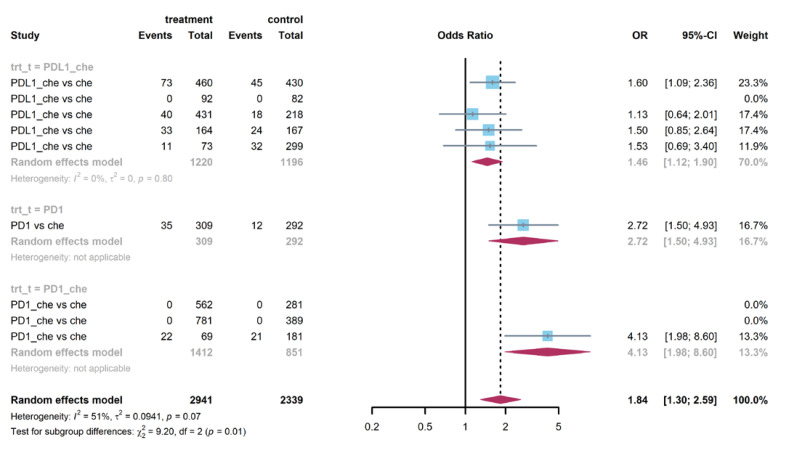
Forest plot of non-serious pruritus in patients treated with PD-1 or PD-L1 inhibitors versus chemotherapy.

As is shown in Figure 5, patients treated with PD-1 or PD-L1 inhibitors were more likely to experience hypothyroidism (OR = 3.63, 95% CI: 2.92–4.51). This trend was clear in both PD-1 groups (OR = 5.74, 95% CI: 1.48–22.20) and PD-L1 groups (OR = 3.85, 95% CI: 2.72–5.44). Compared with patients treated in the control arms, those treated with PD-1 or PD-L1 inhibitors were at higher risk of AST elevation (OR =1.26, 95% CI: 1.02–1.57, see Figure S3). Further analysis showed that there was no report of AST elevation in PD-1 groups. Patients in PD-L1 groups were prone to AST elevation (OR = 1.30, 95% CI: 1.03–1.65). Figure 6 and Figure S4 show that patients were more likely to report pruritus (OR = 1.84, 95% CI: 1.30–2.59) and rash (OR = 1.29, 95% CI: 1.08–1.55) in treatment arms compared with patients in the control arms. Figure S5 demonstrates that PD-L1 combined with chemotherapy groups were at increased risk of fever (OR = 2.04, 95% CI: 1.33–3.14) than the control group.

## 4. Discussion

Although PD-1 or PD-L1 drugs are widely used in lung cancer and melanoma, research on their impacts on triple-negative breast cancer, a refractory breast tumor, is still very limited, as is research on their safety. Several meta-analysis results showed that PD-1 or PD-L1 inhibitors plus chemotherapy prolonged the progression-free survival in the neoadjuvant and adjuvant settings when treating TNBC patients [20,33,34]. In this meta-analysis, we first explored the incidence of adverse events in TNBC patients through the proportion meta-analysis method, and then conducted a traditional meta-analysis to compare the risk of different adverse events between the PD-1 or PD-L1 inhibitors group and the chemotherapy group. We found that, for serious adverse events, neutropenia had the highest incidence, followed by fatigue and anemia. This was consistent with the results from Zhou et al. showing that the common treatment-related adverse events in PD-1 or PD-L1 inhibitors and chemotherapy combination was anemia (45.4%) of all-grade adverse events and neutropenia (19.6%) of grade 3 or higher [21]. The difference is that our meta-analysis did not show any significant results in blood-related adverse events such as neutropenia and anemia. From the results of Keynote-119, the pembrolizumab group had less frequent anemia and neutropenia [27]. In our analysis, eight out of the nine studies used PD-1 or PD-L1 inhibitors combined with chemotherapy for TNBC patients, indicating that the blood toxicity may have been mainly related to chemotherapy.

The most common adverse events were related to digestive reactions, such as nausea (49.7%), diarrhea (32.8%), constipation (23.2%), and vomiting (21.4%). Previous studies have suggested that gastrointestinal-tract-related reactions are the most common all-grade adverse events with anti-PD-1/PD-L1 agents [35,36]. However, our meta-analysis showed that there were no significant results of gastrointestinal reactions (nausea: OR = 1, 95% CI: 0.78–1.29; diarrhea: OR = 1.04, 95% CI: 0.63–1.72; constipation: OR = 1.03, 95% CI: 0.88–1.21;) upon comparing the PD-1 or PD-L1 groups with chemotherapy groups. Doctors should also pay attention to these less severe adverse events and take precautions, since they could affect patient quality of life.

Among the immune-related adverse events, the incidences of the most serious adverse events were very low (less than 1%). For non-serious adverse events, the most common one was arthralgia (24.9%), followed by ALT elevation (18.1%), AST elevation (17.9%), rash (17.6%), and pruritus (15.7%). Previous clinical trials have reported high incidence of immune-related adverse events, up to 58.7% in Impassion130 [23,24,25,27]. In our analysis, pneumonitis, hypothyroidism and hyperthyroidism were less common, but they were more likely to be severe. Compared with chemotherapy, PD-1 or PD-L1 inhibitors had more risk of serious pneumonitis and hypothyroidism, which are similar results to the meta-analyses by Zhang et al. and Wang et al. [33,34]. In the tumor microenvironment, tumor immune escape is related to the role of PD-1/PD-L1 and T lymphocytes [37]. Therefore, immune checkpoint inhibition therapy could affect the balance between autoimmunity and immunity, and thus enhance the activity of the immune system and attack tumor cells [38]. These immune-related adverse events may be due to the immunosuppressive effect of the drugs and should be treated seriously [39].

It is worth noting that, among the serious immune-related adverse events, adrenal insufficiency (1.7%) was very common and the incidence of non-serious adrenal insufficiency was 1.1%. In a study by Wang et al., the incidence of all-grade adverse events was 0.69%, but they did not distinguish between cancer types [22]. In addition, the risk of adrenal insufficiency was significantly higher in the PD-1 or PD-L1 groups than in the chemotherapy groups. Adrenal insufficiency may have a great influence on patient metabolism [36]. Due to diagnostic techniques, doctors may not notice adrenal insufficiency, leading to low incidence rates [40]. Once patients are diagnosed with adrenal insufficiency, it can be so serious that hospitalization is required [41]. Thus, for future TNBC patients treated with PD-1 or PD-L1 inhibitors, adrenal insufficiency should be taken into more consideration.

We performed subgroup analysis of PD-1 and PD-L1 inhibitors. Apart from one study which used PD-1 inhibitors alone, other studies all combined PD-1 or PD-L1 inhibitors with chemotherapy. The overall trends of adverse events were consistent between the PD-1 group and the PD-L1 group, though the data were limited and some adverse events may not have been reported in some studies. Sonpavde et al.’s study showed that the incidence of grade 3 or higher adverse events was higher in the PD-1 inhibitors group compared with PD-L1 inhibitors [42]. Campelo et al. found that PD-L1 inhibitors were associated with a lower risk of adverse events that led to treatment discontinuation than PD-1 inhibitors [43]. The use of PD-1 or PD-L1 inhibitors significantly increased the risk of serious immune-related adverse events in this study [43], but the incidences were very low. Meanwhile, the risk of non-serious adverse events did not increase. Considering its efficacy, we consider the safety of PD-1 or PD-L1 inhibitors as acceptable. However, adverse events were not the primary outcome of the included studies. We cannot avoid incomplete data reports even if we extract the data from the clinical trial registration website [44]. More research on PD-1 or PD-L1 inhibitors in TNBC patients is needed.

The limitations of our study are as follows. First, we used adverse event data from clinicaltrial.gov. For those studies that had no data on this website, we extracted data from their published articles. Heterogeneity may arise from these different data sources [45]. Second, we only included II/III randomized clinical trials in our meta-analysis. The number of studies was still limited. In the future, to investigate the safety of PD-1 or PD-L1 Inhibitors more comprehensively, some observational studies such as cohort studies may also be considered. Third, we did not distinguish between chemotherapy regimens, doses of PD-1 or PD-L1 inhibitors, patient age, etc. Lastly, because the incidence of serious adverse events was usually very low, regular meta-analysis approaches were not applicable, which leads to the challenge of rare event modeling [46]. In this study, we adopted the Peto method to tackle this problem. Other meta-analysis methods for rare events may also be applicable to this study.

## 5. Conclusions

Compared to the treatment of chemotherapy alone, PD-1 or PD-L1 inhibitors combined with chemotherapy significantly increased the risk of immune-related adverse events in TNBC patients, including serious pneumonitis, hypothyroidism, and adrenal insufficiency, but the incidences were relatively low. For practical treatment using PD-1 or PD-L1 inhibitors in TNBC, serious adverse events, such as serious pneumonitis, hypothyroidism, ALT elevation, and adrenal insufficiency, should be considered and monitored. Non-serious adverse events, such as AST elevation, rash, and fever, should also be taken into consideration.

## Figures and Tables

**Table 1:** Characteristics of all the included studies.

Year	Title	Authors	NCT Number	Study	Treatment
2021	First-line atezolizumab plus nab-paclitaxel for unresectable, locally advanced, or metastatic triple-negative breast cancer: IMpassion130 final overall survival analysis	Emens, L.A., et al.	NCT02425891	IMpassion130	Atezolizumab+ chemotherapy
2021	Pembrolizumab versus investigator-choice chemotherapy for metastatic triple-negative breast cancer (KEYNOTE-119): a randomized, open-label, phase 3 trial	Winer, E.P., et al.	NCT02555657	KEYNOTE-119	Pembrolizumab
2019	A randomized phase II study investigating durvalumab in addition to an anthracycline taxane-based neoadjuvant therapy in early triple-negative breast cancer: clinical results and biomarker analysis of GeparNuevo study	Loibl, S., et al.	NCT02685059	GeparNuevo	Durvalumab+ chemotherapy
2020	Pembrolizumab plus chemotherapy versus placebo plus chemotherapy for previously untreated locally recurrent inoperable or metastatic triple-negative breast cancer (KEYNOTE-355): a randomized, placebo-controlled, double-blind, phase 3 clinical trial	Cescon, D., et al.	NCT02819518	KEYNOTE-355	Pembrolizumab+ chemotherapy
2020	Pembrolizumab for early triple-negative breast cancer	Schmid, P., et al.	NCT03036488	KEYNOTE522	Pembrolizumab+ chemotherapy
2021	Primary results from IMpassion131, a double-blind, placebo-controlled, randomized phase III trial of first-line paclitaxel with or without atezolizumab for unresectable locally advanced/metastatic triple-negative breast cancer	Miles, D., et al.	NCT03125902	IMpassion131	Atezolizumab+ chemotherapy
2020	Neoadjuvant atezolizumab in combination with sequential nab-paclitaxel and anthracycline-based chemotherapy versus placebo and chemotherapy in patients with early-stage triple-negative breast cancer (IMpassion031): a randomized, double-blind, phase 3 trial	Mittendorf, E, et al.	NCT03197935	IMpassion031	Atezolizumab+ chemotherapy
2021	Durvalumab with olaparib and paclitaxel for high-risk HER2-negative stage II/III breast cancer: Results from the adaptively randomized I-SPY2 trial	Pusztai, L., et al.	-	-	Durvalumab+ chemotherapy
2020	Effect of pembrolizumab plus neoadjuvant chemotherapy on pathologic complete response in women with early-stage breast cancer an analysis of the ongoing phase 2 adaptively randomized I-SPY2 trial	Nanda, R., et al.	NCT01042379	-	Pembrolizumab+ chemotherapy

**Table 2:** Risk of bias assessment of included studies.

Study	Randomization Process	Deviations from Intended Interventions	Missing Outcome Data	Measurement of the Outcome	Selection of the Reported Result
Emens, L.A., 2021 [23]	low	low	low	low	low
Winer, E.P., 2021 [27]	low	low	low	low	low
Loibl, S., 2019 [32]	unclear	low	low	low	low
Cescon, D., 2020 [25]	low	low	low	low	low
Schmid, P., 2020 [29]	low	low	low	low	low
Miles, D., 2021 [24]	low	low	low	low	low
Mittendorf, E., 2020 [30]	low	low	low	low	low
Pusztai, L., 2021 [31]	unclear	high	unclear	high	low
Nanda, R., 2020 [28]	unclear	high	unclear	low	low

## Supplementary Materials

The following supporting information can be downloaded at: https://www.mdpi.com/article/10.3390/life12121990/s1, **Figure S1**: Forest plot of serious hypothyroidism in patients treated with PD-1 or PD-L1 inhibitors versus chemotherapy; Figure S2: Forest plot of serious adrenal insufficiency in patients treated with PD-1 or PD-L1 inhibitors versus chemotherapy; Figure S3: Forest plot of other AST elevation in patients treated with PD-1 or PD-L1 inhibitors versus chemotherapy; Figure S4: Forest plot of other rashes in patients treated with PD-1 or PD-L1 inhibitors versus chemotherapy; Figure S5: Forest plot of other fevers in patients treated with PD-1 or PD-L1 inhibitors versus chemotherapy.
