# Supplementary material for: Adverse Events of PD-1 or PD-L1 Inhibitors in Triple-Negative Breast Cancer: A Systematic Review and Meta-Analysis
Source: Life (Basel). 2022 Nov 28;12(12):1990. doi: 10.3390/life12121990 (PMC9787874; doi:10.3390/life12121990)
Supplement: Supplementary file 1 [file life-12-01990-s001.zip › life-2019021-SI.pdf]

Supplementary Material

This supplementary file presents five forest plots illustrating the ORs of the serious hypothyroid, serious adrenal insufficiency, non-serious AST elevation, non-serious rash, and the non-serious fever, respectively, in patients treated with PD-1 or PD-L1 inhibitors versus the patients treated with chemotherapy. In particular, Figure S1 shows the forest plot of serious hypothyroid in patients treated with PD-1 or PD-L1 inhibitors versus chemotherapy; Figure S2 presents the forest plot of serious adrenal insufficiency in patients; Figure S3 displays the forest plot of non-severe AST elevation in patients; Figure S4 shows the forest plot of other rash in patients; and Figure S5 presents the forest plot of non-severe fever in patients.

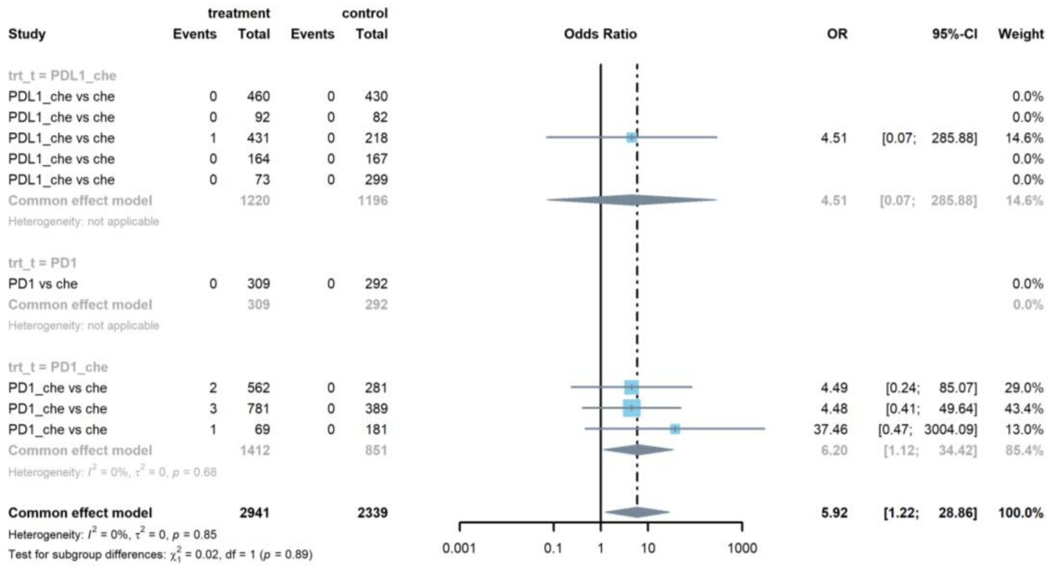

Figure S1. Forest plot of serious hypothyroidism in patients treated with PD-1 or PD-L1 inhibitors versus chemotherapy.

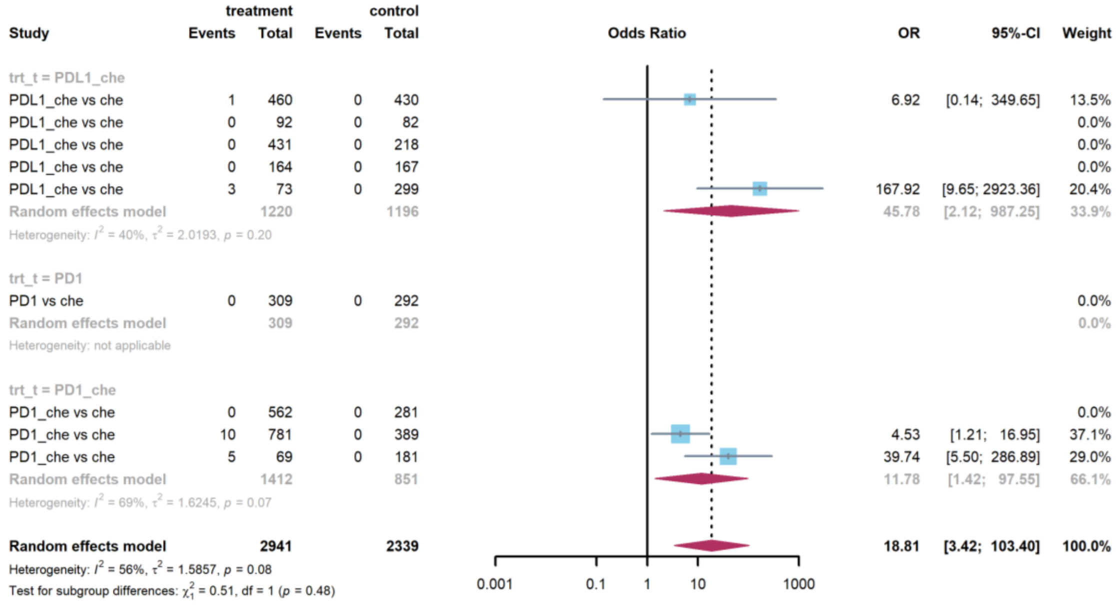

Figure S2. Forest plot of serious adrenal insufficiency in patients treated with PD-1 or PD-L1 inhibitors versus chemotherapy.

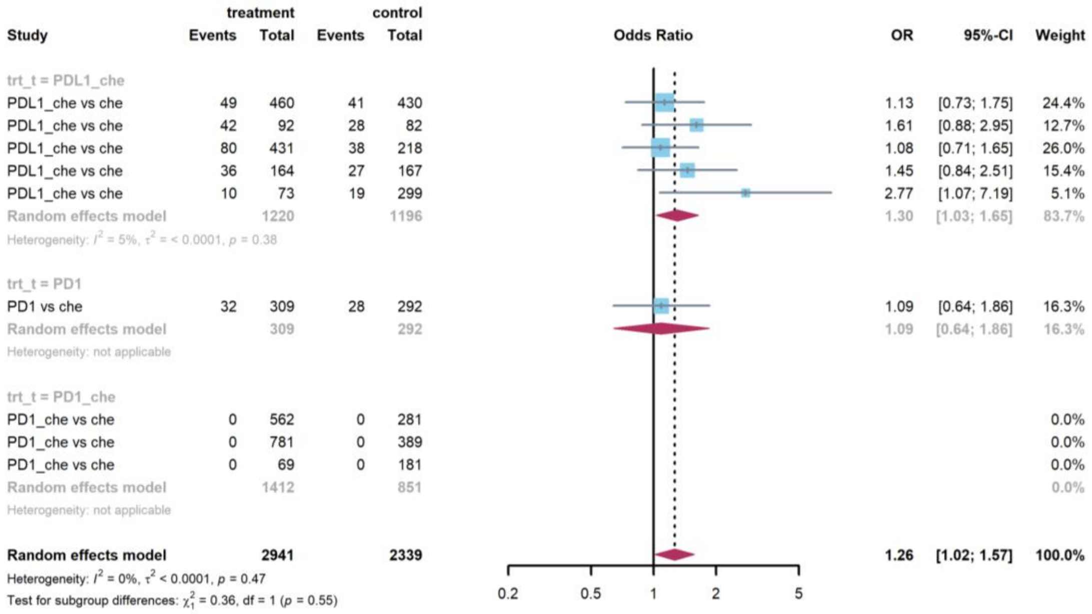

Figure S3. Forest plot of other AST elevation in patients treated with PD-1 or PD-L1 inhibitors versus chemotherapy.

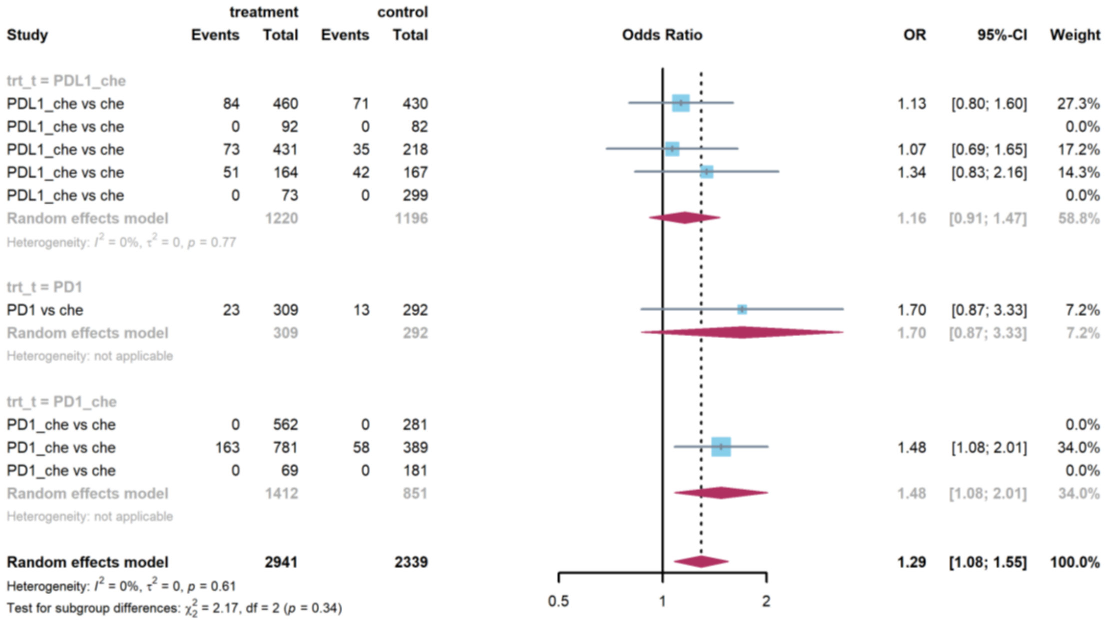

Figure S4. Forest plot of other rashes in patients treated with PD-1 or PD-L1 inhibitors versus chemotherapy.

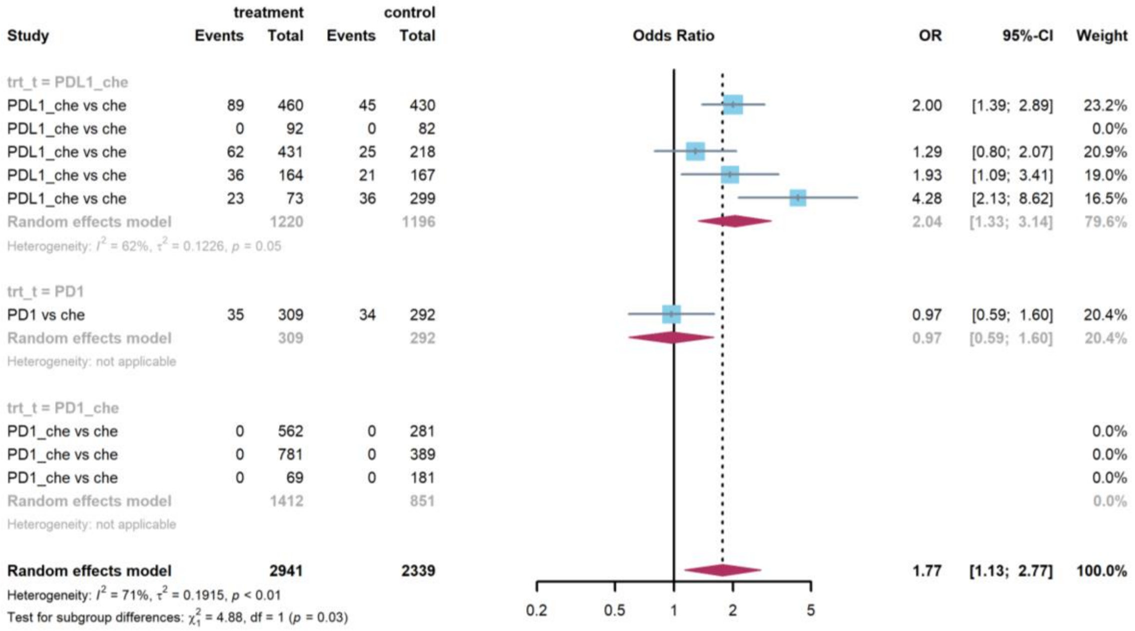

Figure S5. Forest plot of other fevers in patients treated with PD-1 or PD-L1 inhibitors versus chemotherapy.
